# Analysis of alterations in the composition of the intestinal microbiota in frail older individuals

**DOI:** 10.1371/journal.pone.0320918

**Published:** 2025-05-08

**Authors:** Chuan Zhang, Lu Gong, Shilan Luo, Lamei Yang, Xiaoli Yan

**Affiliations:** 1 Department of Cardiology, The First Affiliated Hospital of Chongqing Medical University, Chongqing, China; 2 Department of Dermatology, The First Affiliated Hospital of Chongqing Medical University, Chongqing, China; 3 Department of Geriatrics, The Second Affiliated Hospital of Chongqing Medical University, Chongqing, China; 4 Health Management Center, The First Affiliated Hospital of Chongqing Medical University, Chongqing, China; Instituto Nacional de Geriatria, MEXICO

## Abstract

**Background:**

Frailty is an ageing-associated geriatric syndrome that severely affects the functional status, quality of life and life expectancy of older adults. Immune dysfunction and chronic inflammation play crucial roles in frailty, and this study aimed to explore the correlation between the intestinal microbiota and frailty.

**Methods:**

A cross-sectional survey was conducted using a comprehensive geriatric assessment of older individuals who underwent medical checkups at the Health Management Center from April 2023 to May 2024. A total of 672 older individuals who met the inclusion criteria were included and divided into a healthy control group and a frail case group. Clinical data, as well as blood and stool samples, were collected. The data from the two groups were analysed with 16S rRNA sequencing in 20 and 30 cases, respectively. SPSS 25.0 was used for statistical analysis.

**Results:**

There were significant differences in income, smoking, and globulin levels between the two groups, while there were no differences in age or sex. There was no significant difference in the abundance or species evenness of intestinal bacteria between the two groups. However, the abundance of accessory bacteria, bifidobacteria, and Escherichia coli in the frail group was greater than that in the control group. Specifically, Escherichia-Shigella was significantly upregulated and fit well into the prediction model of frailty.

**Conclusion:**

The gut microbiota of frail older individuals underwent significant changes in structural composition, and the presence of Escherichia-Shigella may be a diagnostic marker for debilitating diseases. These findings provide an essential clinical reference value for developing methods for preventing or alleviating frailty based on specific microbial communities.

## Introduction

Frailty is an ageing-related geriatric syndrome in which the body exhibits a decline in physiological reserve function, reduced stress resistance, and attenuation of muscular strength and endurance as ageing progresses [1]. Epidemiological studies have shown that 7% of people older than 65 are debilitated and that 20% of individuals older than 80 are frail [2]. Frailty is a multidimensional indicator of ageing and reflects the cumulative decline of multiple physiological systems, as it involves multisystem physiological changes in the neuromuscular system, metabolism, and immune system [3,4]. Frailty manifests not only as the weakening of somatic functions but also as the disruption of endoenvironmental functions, which in turn leads to adverse events such as falls, disability, and even death. It is necessary to identify and manage the pathogenic factors that cause weakness.

Previous studies have shown that older age, poorer economic conditions, lower levels of education, unhealthy lifestyles such as smoking and alcohol consumption, unmarried status and living alone may promote frailty development [1,3,4]. However, the pathophysiology of frailty has not been fully elucidated. Nevertheless, some studies have shown that immune dysfunction and chronic inflammation play critical roles in frailty [5,6]. It has been suggested that frailty-associated gut bacterial dysbiosis leads to impaired intestinal barrier function and chronic inflammation, a process that involves metabolic disruption and may be a key step in the development of frailty [7]. Since gut bacteria are associated with immune function and chronic inflammation, the correlation between gut bacteria and frailty has gained increasing attention in recent years. Gut microorganisms are complex and diverse microbiota living in the human gut in symbiosis with humans and are closely related to human physiology, metabolism, disease, and ageing. Dysbiosis of the gut microbiota, also known as intestinal flora disorder, is caused primarily by changes in the composition of the gut microbiota, bacterial activity, or the distribution of the microbiota. Alterations in the composition and function of the gut microbiota have direct effects on human health and play crucial roles in the occurrence of several diseases, such as inflammatory bowel disease, T2DM, obesity, cardiovascular disease and neurodegenerative diseases [8]. Dysbiosis of the gut microbiota can also lead to the occurrence of frailty. In addition to chronic inflammation and the immune response, gut microbes may also promote frailty development through oxidative stress, metabolic processes, brain-gut-axis regulation, etc. [9–11]. Several studies have shown that the composition of gut microbes in frail older individuals is different from that in healthy older individuals and that multiple bacterial genera are different between frail and healthy older individuals [12,13]. For example, Enterobacteriaceae may be associated with a higher frailty index, whereas Oscillospira and Ruminococcus are enriched in sarcopenic and frail subjects [14,15]. A study involving 1448 older individuals revealed significant differences in the overall community structure of gut bacteria among different frailty groups. The degree of deviation in bacterial structure between the frail and non-frail groups gradually increased with increasing severity of frailty. The severity of frailty explained more of the variation in the bacterial community structure of the older individuals than did demographic factors such as age and sex; clinical, physiological, and biochemical factors such as blood pressure and blood glucose; and various lifestyle or health indicators. The authors also performed a validation analysis using a metagenomic dataset, which revealed that the relationship between the abundance of nine gut bacteria and the impact of frailty on older individuals could be reproduced well enough to determine the effects of gut bacteria on the frailty status of older individuals [16]. In recent years, owing to advances in gene sequencing technology, more studies have shown that the gut microbiome is significantly correlated with frailty and have demonstrated frailty-associated gut bacterial characteristics [17]. Moreover, gut bacteria can be modulated through diet, prebiotic, and probiotic interventions to alleviate overall symptoms of frailty, including decreased exercise tolerance, fatigue, muscle weakness, etc. [18,19].

Here, we analysed the changes in serum biochemical indicators, such as relevant vitamins, proteins and some trace elements, between frail and healthy older individuals. Moreover, we characterised and compared healthy and frail older individuals using 16S rRNA gene sequencing, discussed the gut microbiota characteristics associated with frailty in older individuals, and searched for microbial markers that might serve as markers of frailty in this population.

## Materials and methods

### Study population

From April 1, 2023 to May 30, 2024, a cross-sectional survey was conducted on older individuals who underwent medical checkups at the Health Management Centre of the First Affiliated Hospital of Chongqing Medical University. The inclusion criteria were as follows: 1. met the criteria for frailty in older individuals; 2. aged 60–85 years; 3. had normal cognitive function and the ability to complete the physical assessment test; and 4. voluntarily participated in this study and signed an informed consent form. The exclusion criteria were as follows: 1. combined cardiovascular, cerebrovascular, hepatic, renal, pulmonary or haematopoietic system and other serious diseases; 2. a combination of malignant tumours, gastrointestinal diseases, diabetes mellitus, active pulmonary tuberculosis and other diseases that seriously affect nutritional status; 3. Use of probiotics and traditional Chinese medicine; and 4. participation in other interventional trials. This study was approved by the Medical Ethics Committee of the First Affiliated Hospital of Chongqing Medical University (Ethics approval number: k2024-051–01, date approved: January 26, 2024) in Chongqing, China, and all participants provided written informed consent.

### Clinical information and blood and stool sample collection

All participants fasted for eight hours, after which serum was collected early the following morning. Faecal samples were collected via faecal collection tubes. The following general information was collected via questionnaires or electronic medical records: 1. general information, such as age, sex, number of medications taken, smoking history, history of alcohol consumption, income, hospitalisation, and residence; 2. blood indicators including nutritional indicators, such as total protein, haemoglobin and total haemoglobin, albumin, globulin, vitamin D, alanine aminotransferase, aspartate aminotransferase, creatinine and uric acid; and glycolipid metabolism indicators, such as fasting blood glucose, total cholesterol, triacylglycerols(triglycerides, TG), high-density lipoprotein, and low-density lipoprotein; and electrolyte indicators, such as calcium, magnesium, and phosphorus. The height, body mass, waist circumference, hip circumference, blood pressure, pulse rate, grip strength and walking speed of the study subjects were measured according to a unified standard method, and BMI was calculated. Routine blood samples were analysed with a blood cell analyser (Myeri, BC-6600P-1) and a chemiluminescence analyser (Abbott, C16000 HX-009) for biochemical indices.

### Comprehensive assessment of ageing

Fried’s frailty phenotype: In Fried’s frailty phenotype model, individuals have three or more of the following five phenotypes: weak grip strength, low energy expenditure, slow gait speed, self-imposed exhaustion (self-reported feelings of fatigue), and weight loss [20]. According to Fried’s frailty phenotype assessment method, older individuals can be categorised as healthy, prefrail, or frail older individuals. In this study, information on relevant indicators in frail and healthy older individuals was obtained.

### DNA extraction and sequencing

Total DNA was extracted from 0.5 g of each sample using a Fast DNA Spin Kit for soil (MP Biomedicals, CA, United States), after which the purity and concentration of the DNA were determined by agarose gel electrophoresis. An appropriate amount of the sample was transferred to a centrifuge tube, and the sample was diluted to 1 ng/μl with sterile water. The diluted genomic DNA was used as a template, and the primers specific to the sequencing region were used according to the selected sequencing region. Phusion® High-Fidelity PCR Master Mix with GC Buffer was obtained from England Biolabs. High-efficiency and high-fidelity enzymes were used for PCR. The primers used corresponded to the following regions: the primer for the 16S V4 region was 515F-806R; the primer for the 16S V3-V4 region was 338F-806R; the 18S V4 region primer was 528F-706R; the 18S V9 region primer was 1380F-1510R; the ITS1 region primer was ITS1F-ITS2; and the ITS2 region primer was ITS2-3F-ITS2-4R. Aliquots were mixed according to the concentration of the PCR products, which were detected by 2% agarose gel electrophoresis after sufficient mixing. The products were recovered using a GeneJET Gel Recovery Kit from Thermo Scientific. The library was constructed using the NEBNext® Ultra™ DNA Library Prep Kit for Illumina from New England Biolabs. The constructed library was subjected to Qubit quantification and library testing, and after passing the test, it was subjected to online sequencing via MiSeq.

### Bioinformatics analysis

A certain percentage of dirty data exists within the raw data obtained from sequencing. To increase the accuracy and reliability of the results of the information analysis, the raw data are first filtered according to the sequence quality score to obtain clean data. Splicing was performed according to the overlap situation between double-ended reads. Then, the sequence noise reduction algorithm DADA2 was used to optimise the data and select the taxonomic feature sequences to generate amplicon sequence variants (ASVs) that represent the sequence and abundance information. Species classification analysis was then performed according to the ASVs to obtain the corresponding taxonomic annotations for each ASV, thus obtaining the basic abundance of ASVs and taxonomic lineages for each sample. Moreover, diversity index calculations and community diversity difference tests were performed based on the abundance information of the ASVs, and the community structure was statistically analysed at each taxonomic level. A series of statistical or visualisation analyses based on ASVs, correlation analyses of species composition, phylogenetic analyses, and functional prediction analyses was further performed to determine the microbial community differences among the samples.

### Methods of statistical analysis

SPSS 25.0 was used for data analysis. Quantitative data are presented as the mean ± standard deviation (mean ± SD) or median (P25-P75) according to the Kolmogorov‒Smirnov normal distribution test, and comparisons between groups were made using independent samples *t* test or nonparametric Mann‒Whitney U test (for nonnormal data). Qualitative data such as sex are presented as [n, %], and comparisons between groups were made using the chi-square or Fisher’s exact tests. *P* < 0.05 was considered statistically significant.

## Results

### Participant characteristics

Between April 2023 and May 2024, 672 older adults (average age 68.11 ± 7.17 years) were evaluated for frailty, and routine clinical parameters were measured according to the inclusion and exclusion criteria. A summary of the participant demographics and clinical data can be found in S1 Table. Overall, 481 individuals (average age of 67.78 ± 7.02 years) were in the healthy control group, and 191 (68.94 ± 7.50 years) were in the frail group. There was no significant difference in age (P = 0.058) or sex between the two groups. In terms of income, smoking, alcohol consumption, and hospitalisation, significant differences were found between the frail group and the healthy control group (P < 0.05). For the biochemical indicators, there was a significant difference in globulin levels between the two groups (P < 0.05). Subsequently, we randomly screened 20 and 30 cases in the healthy control and frail groups, respectively, for 16S rRNA sequencing. The fifty participants selected had a mean age of 78.74 (±1.85) years. The demographic characteristics and clinical parameters of the participants are summarised in Table 1. No differences in sex or age were observed between the two groups. According to the significant difference analysis, no significant differences between the two groups were observed in the relevant demographic characteristics or clinical parameters. The changes in the gut microbiota composition between the two groups were subsequently explored.

**Table 1:** Clinical parameters of older adults (n = 50) with cases (frail) and healthy controls.

Clinical parameters	Total (n = 50)	Control (n = 20)	Case (n = 30)	Statistic	*P*
Age, Mean ± SD	78.74 ± 1.85	78.45 ± 1.15	78.93 ± 2.20	t = -1.02	0.315
Gender, n (%)				χ² = 0.23	0.630
Female	32 (64.00)	12 (60.00)	20 (66.67)		
Male	18 (36.00)	8 (40.00)	10 (33.33)		
Smoking, n (%)				χ² = 1.06	0.304
No	36 (72.00)	16 (80.00)	20 (66.67)		
Yes	14 (28.00)	4 (20.00)	10 (33.33)		
Drinking, n (%)				χ² = 2.79	0.095
No	36 (72.00)	17 (85.00)	19 (63.33)		
Yes	14 (28.00)	3 (15.00)	11 (36.67)		
Take multiple medications, n (%)				χ² = 0.95	0.328
No	6 (12.00)	4 (20.00)	2 (6.67)		
Yes	44 (88.00)	16 (80.00)	28 (93.33)		
BMI, Mean ± SD	23.51 ± 2.81	23.91 ± 2.55	23.24 ± 2.98	t = 0.83	0.408
Waistline (cm), Mean ± SD	82.34 ± 8.35	82.75 ± 8.52	82.07 ± 8.37	t = 0.28	0.780
Hip circumference (cm), Mean ± SD	91.74 ± 4.28	92.70 ± 3.20	91.10 ± 4.82	t = 1.30	0.198
Systolic blood pressure (mm/Hg), Mean ± SD	145.70 ± 19.39	146.65 ± 21.66	145.07 ± 18.08	t = 0.28	0.781
Diastolic blood pressure (mm/Hg), Mean ± SD	74.56 ± 8.99	73.10 ± 7.73	75.53 ± 9.75	t = -0.94	0.354
Pulse (times/min), Mean ± SD	72.90 ± 11.05	75.30 ± 11.59	71.30 ± 10.57	t = 1.26	0.213
Vitamin D (ng/ml), Mean ± SD	26.68 ± 10.67	26.75 ± 11.89	26.63 ± 9.98	t = 0.04	0.971
Haemoglobin (g/L), Mean ± SD	132.56 ± 13.46	128.85 ± 12.14	135.03 ± 13.91	t = -1.62	0.112
Total protein (g/L), Mean ± SD	71.84 ± 4.52	71.50 ± 3.85	72.07 ± 4.98	t = -0.43	0.669
Albumin (g/L), Mean ± SD	43.02 ± 2.31	43.35 ± 1.93	42.80 ± 2.54	t = 0.82	0.415
Globulin (g/L), Mean ± SD	28.88 ± 3.53	28.15 ± 3.77	29.37 ± 3.34	t = -1.20	0.237
Total bilirubin (g/L), Mean ± SD	18.11 ± 4.02	19.12 ± 4.76	17.44 ± 3.37	t = 1.46	0.150
Alanine aminotransferase (U/L), M (Q₁, Q₃)	14.00 (11.00, 19.00)	13.00 (11.00, 16.75)	14.00 (12.00, 19.00)	Z = -0.86	0.388
Aspartate aminotransaminase (U/L), M (Q₁, Q₃)	20.00 (17.00, 23.00)	21.00 (16.75, 24.00)	18.50 (17.00, 23.00)	Z = -0.69	0.493
Creatinine (mmol/L), M (Q₁, Q₃)	69.00 (64.25, 80.50)	70.50 (65.00, 81.50)	68.50 (63.25, 79.00)	Z = -0.01	0.992
Uric acid (mmol/L), M (Q₁, Q₃)	315.00 (287.50, 357.75)	315.50 (265.00, 377.25)	315.00 (293.75, 355.00)	Z = -0.37	0.714
Calcium (mmol/L), Mean ± SD	2.39 ± 0.12	2.38 ± 0.09	2.40 ± 0.14	t = -0.65	0.520
Magnesium (mmol/L), Mean ± SD	0.89 ± 0.05	0.89 ± 0.06	0.89 ± 0.03	t = -0.20	0.845
Phosphorus (mmol/L), Mean ± SD	1.15 ± 0.12	1.15 ± 0.15	1.16 ± 0.09	t = -0.18	0.856
Total cholesterol (mmol/L), Mean ± SD	5.22 ± 1.25	5.09 ± 1.29	5.30 ± 1.24	t = -0.59	0.558
Triglyceride (mmol/L), M (Q₁, Q₃)	1.35 (0.97, 1.74)	1.27 (0.84, 1.55)	1.48 (1.08, 2.08)	Z = -1.91	0.056
High-density lipoprotein (mmol/L), Mean ± SD	1.58 ± 0.36	1.60 ± 0.35	1.56 ± 0.36	t = 0.38	0.707
Low-density lipoprotein (mmol/L), Mean ± SD	2.71 ± 0.94	2.65 ± 1.08	2.75 ± 0.85	t = -0.39	0.696
Fasting blood glucose (mmol/L), Mean ± SD	5.82 ± 1.45	5.85 ± 1.52	5.80 ± 1.42	t = 0.11	0.912

Z: Mann‒Whitney test; χ²: chi-square test; -: Fisher’s exact test; M: Median, Q₁: 1st quartile, Q₃: 3rd quartile.

M (Q₁, Q₃): Median (M), interquartile spacing (P25–P75).

### Alpha analysis

**Fig 1:**
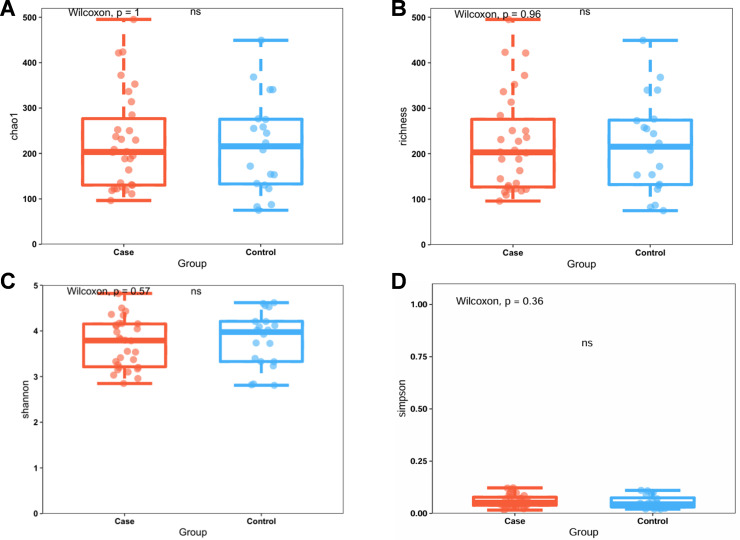
Faecal microbiota alpha diversity indices in older adults with frailty (Case) and health (control). **(A)** Chao1 index. **(B)** Richness index. **(C)** Shannon diversity index. **(D)** Simpson index. Each box plot shows the median, interquartile range, and minimum and maximum values. ns, not significant.

Alpha diversity quantifies the diversity within a specific habitat or ecosystem and is typically assessed through two key metrics: species richness and evenness. Species richness denotes the total number of distinct species present in the community, whereas evenness indicates the uniformity of species distribution within the community. The sparse curves of all samples tended to be flat, which suggests that the data volume is progressive and reasonable and that the sequencing depth meets the requirements of the subsequent analysis (S1A Fig). ANOSIM revealed that the difference between the two groups was significantly greater than the difference within the groups (P < 0.05), suggesting that grouping has practical significance (S1B Fig). Analysis of the collected faecal samples revealed no significant differences in alpha diversity according to the Chao1 index (Fig 1A, P = 1.00), richness index (Fig 1B, P = 0.96), Shannon diversity index (Fig 1C, P = 0.57), or Simpson index (Fig 1D, P = 0.36), which suggests that the richness and uniformity of the gut microbiota in the two groups were not significantly different.

### Beta diversity and functional analysis

**Fig 2:**
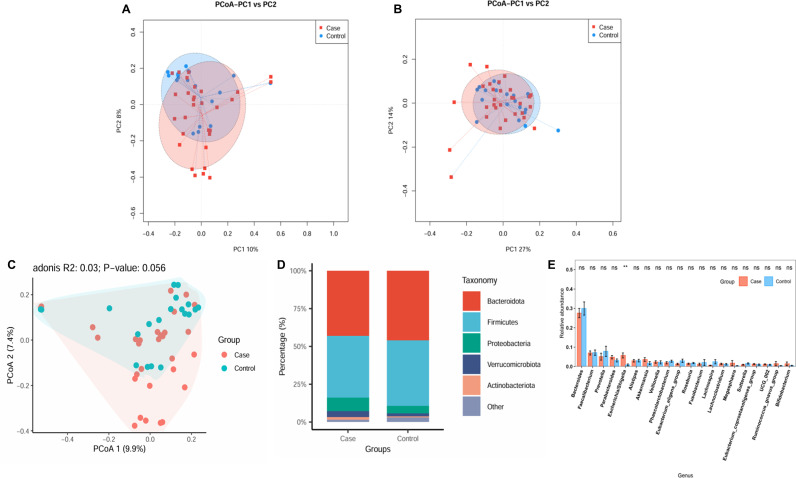
Faecal microbiota beta diversity indices and taxonomy in older adults in the case and control groups. The similarity level of microbial communities detected in the case group (red) and the control group (blue) was evaluated through principal coordinate analysis (PCoA) based on **(A)** Bray–Curtis distances, (B) weighted UniFrac, and **(C)** PCoA2 component analysis. These analyses revealed the top 8 most abundant phyla (D) and the top 20 most abundant genera (E) between the case and control groups. **P < 0.01. ns, not significant.

Beta diversity is primarily utilised to characterise the dissimilarities in species composition across distinct habitats or communities and was characterised for samples via weighted UniFrac (Fig 2A) and Bray‒Curtis distances (Fig 2B). Beta diversity is used to assess the variability in faecal microbiota structures and was characterised for samples via weighted UniFrac (Fig 2A) and Bray‒Curtis distances (Fig 2B). Most samples in the PCoA were distinctly clustered between the frail and control groups. Based on X-matrix principal coordinate analysis, significant differences in microbial composition were observed between the two groups (Fig 2C). The KEGG functions of the two groups were further enriched. The differences were shown in 11 functional regions (S2A and S2B Fig), with a focus on linoleic acid metabolism (p < 0.01), biosynthesis of siderophore group nonribosomal peptides (P < 0.01), and lipid metabolism (P < 0.01), among others.

### Alterations in taxa

We further analysed the specific changes in the gut microbiota of frail participants. The relative abundance accumulation histogram at the phylum level shows the abundance of the top 5 flora in each sample (S1C Fig). Bacteroidetes, Firmicutes, Proteobacteria, Verrucomicrobiota, and Actinobacteriota were identified as the most abundant sequences (Fig 2D). The cluster heatmap shows the relative abundance of the top 30 genera in 50 samples (S1D Fig). Compared with the control group, the frailty group’s relative abundances of Bacteroides, Faecalibacterium, Prevotella, Phascolarctobacterium, Lachnospira and Sutterella were lower. However, the taxa Akkermansia, Megasphaera, and Bifidobacterium levels increased, especially the percentages of Escherichia-Shigella, which increased significantly at the genus level (Fig 2E). The grouped stacked columns show the top 20 most abundant genera between the case and control groups (S1E Fig).

**Fig 3:**
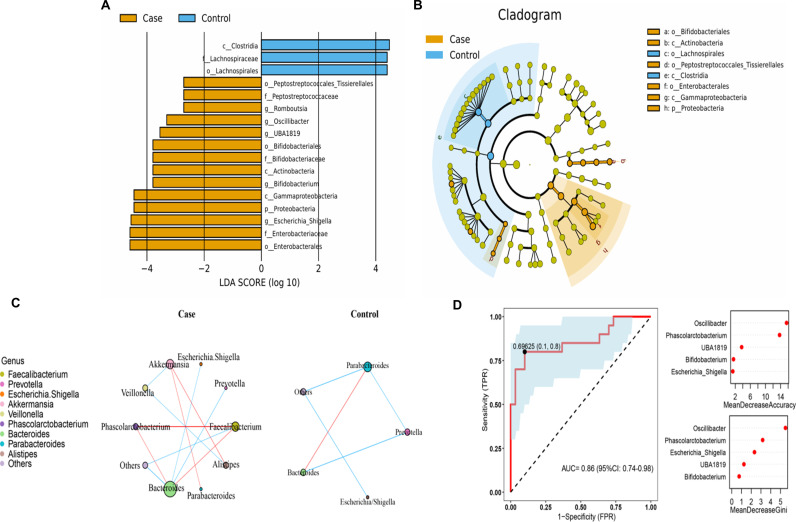
Analysis of differential microbiome and classification prediction model between case and control groups. **(A)** Linear discriminant analysis [LDA; (log10)>2] and (B) effect size (LEfSe) analysis revealed significant differences (P < 0.01) in the microbiota of the case (orange, negative score) and control groups (blue, positive score) groups. **(C)** The distinctive gut microbial coabundance networks between case and control groups. Spearman’s correlation analysis was used to assess the relationships between the top 10 most abundant taxa at the genus level in the frail and control groups. The blue colour indicates that the correlation coefficient is negative, the red colour indicates that the correlation coefficient is positive, and the size of a point is positively correlated with the number of lines connecting that point. **(D)** The random forest (RF) model was employed to construct a predictive model for taxa at the genus level. The receiver operating characteristic (ROC) curve was generated using 5 genera in the faecal microbiota. The relative importance of each genus in the predictive model was evaluated using the mean decreasing accuracy and the Gini coefficient.

LDA and LEfSe analyses were used to identify differences in the gut microbiota between the two groups. Significant differences were observed in the faecal microbiota collected from older, frail adults and healthy controls. The relative abundances of Escherichia-Shigella, Bifidobacterium, UBA1819, Romboutsia, and Oscillibacter were greater in older, frail adults (case group). In contrast, Clostridia, Lachnospiraceae, and Lachnospirals were enriched in the healthy controls (Fig 3A and 3B). The top 10 most abundant genera were further evaluated using Spearman’s correlation analysis (Fig 3C), revealing that intergenus interactions were more common in frail patients than healthy controls.

### Gut microbiota composition can be used to distinguish older frail adults from healthy controls

We applied RF to construct a predictive model to identify the faecal microbiota for sample classification. Combined with the previous analysis of gut microbes, we screened 5 key microorganisms at the level of the top 20 genera. These genera included Oscillibacter, Phascolarctobacterium, UBA1819, Bifidobacterium and Escherichia-Shigella. The mean decrease in accuracy and the Gini coefficient of the faecal microbiota were used to evaluate the relative importance of each genus in the predictive model. We used ROC analysis to assess the accuracy of the sample classifications (Fig 3D). The area under the curve (AUC) was 86% in the specificity and sensitivity analysis.

## Discussion

Some studies have shown that frailty is affected by age, sex, race, income, education, marital status, and some unhealthy habits, such as smoking and alcohol consumption [21,22]. While older age does not necessarily equate to frailty, the prevalence of frailty increases with age, and other studies have suggested that the prevalence of frailty is greater in women than in men [23–25]. Education, marital status, and income may impact a person’s cognition. Impaired cognitive function increases the risk of malnutrition, which is closely associated with frailty and is an intervenable risk factor for frailty [26]. Some biochemical parameters differ between frail and healthy older individuals. For example, haemoglobin may be a protective factor against frailty, and albumin is also correlated with frailty, as both haemoglobin and albumin levels are significantly lower in older frail individuals than in healthy older individuals. Additionally, total protein levels are also lower [27–29]. The results of a health and nutrition survey conducted in the US demonstrated that frail seniors have higher fat levels and larger waistlines [30]. The intestinal flora of older individuals with high animal protein and high fat intake was primarily the Bacteroides intestinal type, with decreased diversity of intestinal flora and increased levels of validation markers [31]. Zhang et al. reported that the reduced abundance of Turicibacter in the frail group may lead to metabolic-related diseases, such as obesity, in old individuals and promote frailty [32].

In our study, we compared relevant clinical parameters between the healthy control group and the frail group according to the inclusion criteria. We found that sex was not significantly correlated with frailty in older individuals and that age may influence the occurrence of frailty to some extent. Poor lifestyle habits such as smoking and alcohol consumption were significantly associated with frailty, whereas income and living conditions were not. The essential trace elements calcium, magnesium and phosphorus were measured, and the levels were not significantly different between the two groups. The levels of haemoglobin, total protein, albumin, and some lipid-metabolising enzymes and metabolites were also not significantly different between the two groups, which differs from the findings of previous studies. Interestingly, we found that the globulin level was significantly greater in frail older individuals than in controls (S1 Table). Increased globulin may be associated with immune dysfunction and enhanced inflammatory response in frail older individuals [33] and may serve as a marker of frailty in the future.

Frailty manifests as multiple metabolic abnormalities of bone, muscle, and energy and involves enhanced inflammatory responses, endocrine dysregulation, and immune dysfunction [6,34]. In addition, gut microbes regulate these processes through the gut–brain axis. Vitamin D plays an essential role in bone health and calcium and phosphorus metabolism, and studies have shown that vitamin D regulates gut microbes [35]. Sarcopenia is a significant component and manifestation of frailty; it is defined as a decrease in muscle mass and function and is a central feature of frailty. Physical frailty and sarcopenia exhibit significant clinical overlap, as they share common pathophysiological mechanisms such as insulin resistance, chronic inflammation, and oxidative stress.

In contrast, the composition of the human gut microbiota changes with age-induced sarcopenia and frailty [36,37]. Sequencing analysis of gut bacteria in older Koreans found significant changes in metabolic and inflammatory biomarkers in frail older individuals compared with healthy controls. In contrast, the abundance of beneficial gut flora decreased, and the abundance of harmful flora increased in the frail population [38]. Several studies have reported reduced gut microbiota diversity and gut permeability in frail populations [14,39]. Other studies imply that frailty is not related to the diversity of gut microbes but is influenced mainly by the gut microbiota’s compositional structure; i.e., the structure of the gut bacteria in frail older individuals gradually deviates from that of healthy older individuals [40]. In this study, to explore the relationship between gut microbiota and frailty, we excluded the interference of unhealthy living habits and malnutrition conditions (Table 1). We subjected 50 patients (clinically frail and healthy older individuals) to 16S rRNA sequencing analysis to explore the differences in the gut microbiota between the two groups. We report no significant difference in the richness of the gut bacteria or the evenness of the species between the two groups. No significant difference was observed in the abundance or species uniformity of the intestinal flora between the two groups, but the difference in the flora composition between the two groups was significant. This conclusion is consistent with the findings of Xu et al. [41]. In their study, predominant genera, such as Escherichia-Shigella, Pyramidobacter, Alistipes, and Akkermansia, were positively correlated with IL-6 in the frail group. Alistipes was found to be positively correlated with HGMB1. Both Alistipes and Akkermansia were positively correlated with zonulin. IL-6 and HGMB1 are serum inflammatory markers, whereas zonulin-1 is a marker of intestinal permeability [42–44].

Although IL-6, HGMB1, and zonulin levels were not measured in our study, we found that the abundance of Escherichia-Shigella was significantly increased in the frail group and that the abundance of Akkermansia tended to increase (Fig 2E), suggesting that an inflammatory response and changes in intestinal permeability may have occurred in the frail group. Additionally, in our study, Parabacteroides and Bifidobacterium tended to be increased in the frail group relative to the healthy control group, which is also consistent with the findings of Xu et al. Related studies have reported that Parabacteroides might play a positive regulatory role in glucose and lipid metabolism and that Bifidobacterium can ameliorate age-related muscle damage [45,46]. Escherichia-Shigella is the causative agent of human bacillary dysentery, commonly known as dysentery bacillus. Intestinal microecological dysregulation characterised by significant expansion of Escherichia-Shigella is also a hallmark of IgA nephropathy and may serve as a diagnostic biomarker and therapeutic target for IgA nephropathy [47]. Early symptoms of Escherichia-Shigella infection include diarrhoea, followed by bacterial invasion of the colonic epithelium, ultimately leading to inflammatory colitis; these are interdependent processes amplified by the local release of cytokines and infiltration of inflammatory factors [48]. In addition, in the prediction model of the frail and healthy groups constructed in this study (Fig 3D), Escherichia-Shigella had a high fit in the prediction model. Based on these findings, it is reasonable to hypothesis that Escherichia-Shigella may serve as a diagnostic marker for frailty syndrome.

In summary, frailty is an evolving process that is also considered a reversible geriatric syndrome, as frailty in older individuals appears to be alleviated by early interventions. Therefore, active early screening to identify effective frailty interventions is critical to improve the quality of life of older individuals and reduce the public health burden. While gut microbes may play an essential role in the evolution of frailty, early assessment of gut microbial changes in frail older individuals may improve their frailty status. In the future, more in-depth studies are needed to demonstrate the impact of the gut microbiota on frailty. Developing personalised microbiome-based strategies to reduce the severity of frailty in older individuals may prevent the onset of frailty in older individuals.

### Limitations

This study had several limitations. Although we had rigorous inclusion and exclusion criteria, we could not completely exclude the influence of certain factors, including diet, hygiene habits, and living environment, among others, that might have affected our results. This is a common challenge when studying the gut microbiota. We attempted to minimise these factors’ influence on the statistical analysis results. In addition, the small sample size used for the sequencing analysis limits the conclusions we can draw. Nevertheless, this initial study provides insights and ideas for subsequent in-depth exploration of the gut microbial markers of frailty.

## Supporting information

S1 Fig Genetic diversity of the intestinal microbiota in frail older individuals. (A) Rarefaction curve: the abscissa represents the number of randomly selected sequences, and the ordinate represents the number of OTUs. Each curve represents a sample, all rarefaction curves reached plateaus, suggesting accurate sequencing depth. (B)The level of similarity between the fecal microbial communities detected in the frail and control groups was assessed using an unweighted Analysis of similarity (ANOSIMs). (C) Stacked histograms of relative abundance of species at the gate level. (D) The clustering heat maps of species relative abundance at the top 30 genera level. (E) The analyses revealed the top 20 most abundant genera between the frail and control groups.
(TIF)

S2 Fig Functional diversity of the intestinal microbiota in frail older individuals. (A) Analysis of the functional differences between the T-test groups. (B) The heat map analysis of functional differences between the two groups.
(TIF)

S1 Table The clinical parameters of older adults in case (frailty) and healthy control groups. (DOCX)

S1 Data Demographic characteristics of the study population and examination results. (XLS)
