# Supplementary figures and images for: Analysis of alterations in the composition of the intestinal microbiota in frail older individuals
Source: PLoS One. 2025 May 8;20(5):e0320918. doi: 10.1371/journal.pone.0320918 (PMC12061151; doi:10.1371/journal.pone.0320918)

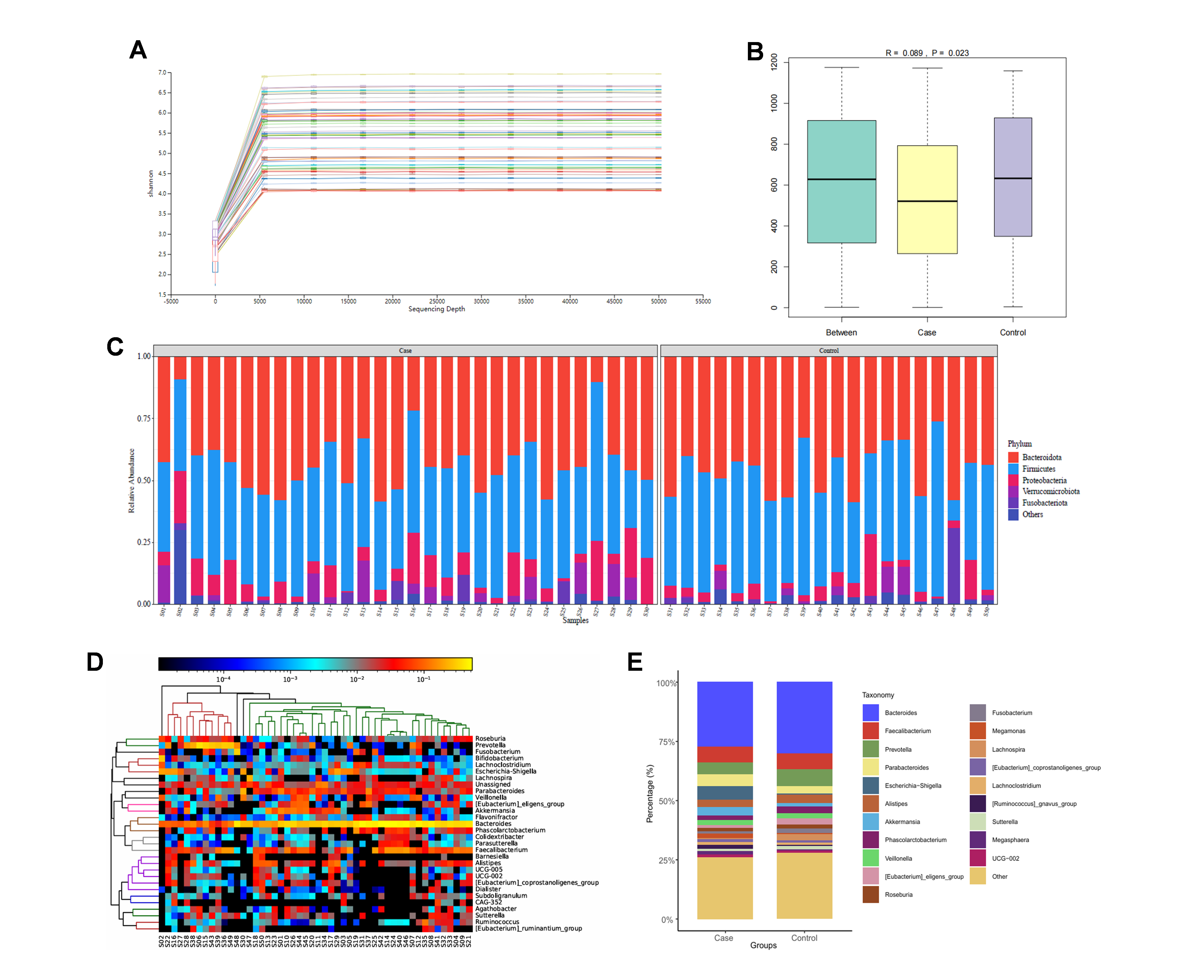

Supplement: S1 Fig — (A) Rarefaction curve: the abscissa represents the number of randomly selected sequences, and the ordinate represents the number of OTUs. Each curve represents a sample, all rarefaction curves reached plateaus, suggesting accurate sequencing depth. (B)The level of similarity between the fecal microbial communities detected in the frail and control groups was assessed using an unweighted Analysis of similarity (ANOSIMs). (C) Stacked histograms of relative abundance of species at the gate level. (D) The clustering heat maps of species relative abundance at the top 30 genera level. (E) The analyses revealed the top 20 most abundant genera between the frail and control groups. (TIF) [file pone.0320918.s001.TIF]

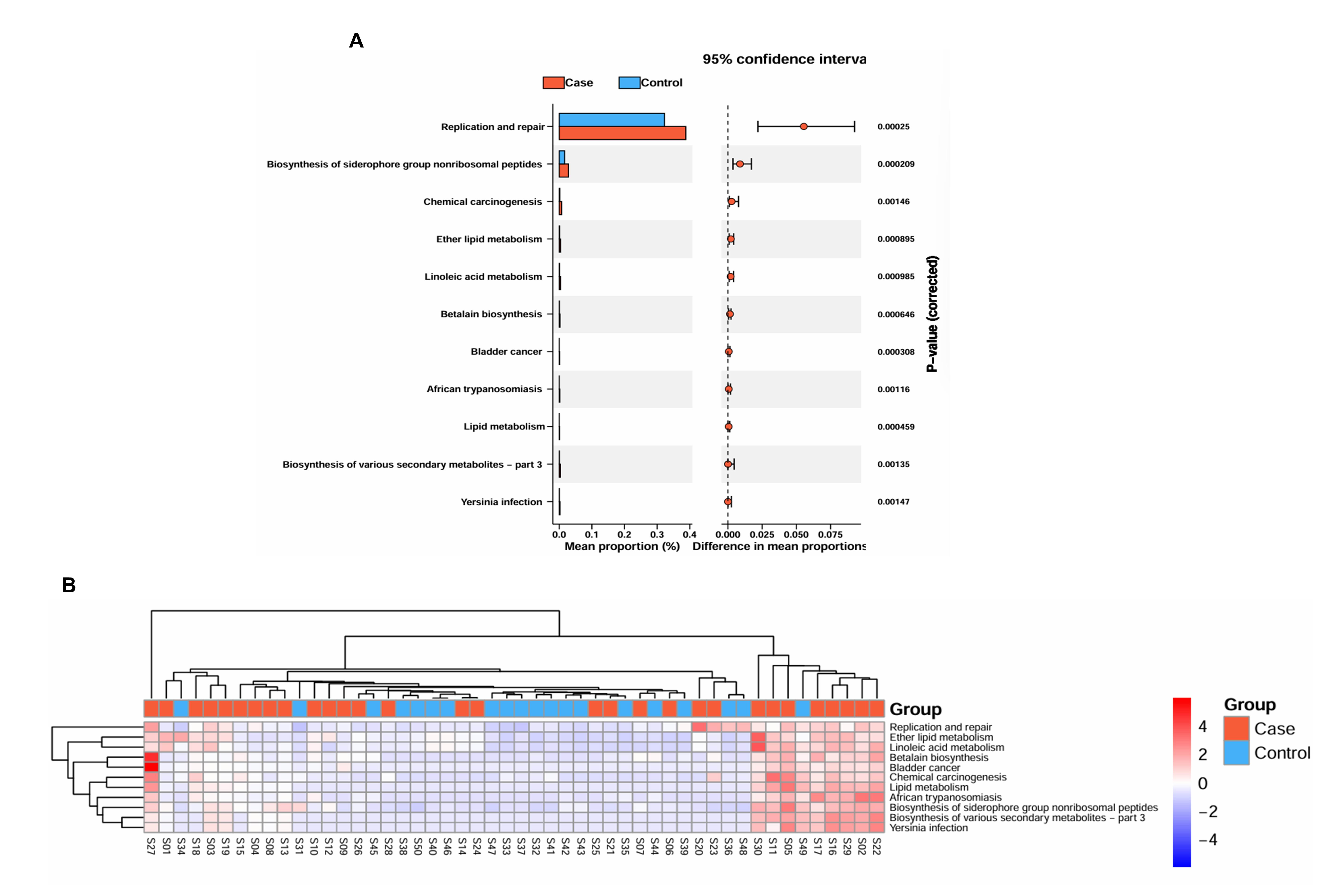

Supplement: S2 Fig — (A) Analysis of the functional differences between the T-test groups. (B) The heat map analysis of functional differences between the two groups. (TIF) [file pone.0320918.s002.TIF]
