# Supplementary material for: Analysis of alterations in the composition of the intestinal microbiota in frail older individuals
Source: PLoS One. 2025 May 8;20(5):e0320918. doi: 10.1371/journal.pone.0320918 (PMC12061151; doi:10.1371/journal.pone.0320918)
Supplement: S1 Table — (DOCX) [file pone.0320918.s003.docx]

Table S1. The clinical parameters of older adults in case (frailty) and healthy control groups.

| **Clinical parameters** | **Total (n = 672)** | **Control (n = 481)** | **Case (n =191)** | **Statistic** | ***P*** |
| --- | --- | --- | --- | --- | --- |
| **Basic Information** | | | | | |
| Age, Mean ± SD | 68.11 ± 7.17 | 67.78 ± 7.02 | 68.94 ± 7.50 | t= -1.90 | 0.058 |
| Gender, n (%) |  |  |  | χ²=0.08 | 0.780 |
| Female | 452 (67.26) | 322 (66.94) | 130 (68.06) |  |  |
| Male | 220 (32.74) | 159 (33.06) | 61 (31.94) |  |  |
| BMI, Mean ± SD | 24.07 ± 3.04 | 24.03 ± 3.02 | 24.16 ± 3.09 | t=-0.52 | 0.605 |
| waistline (cm), Mean ± SD | 83.70 ± 8.94 | 83.65 ± 9.08 | 83.84 ± 8.63 | t=-0.25 | 0.803 |
| Hip circumference (cm), Mean ± SD | 93.04 ± 6.38 | 93.12 ± 5.41 | 92.82 ± 8.34 | t=0.56 | 0.575 |
| Income (Yuan), n (%) |  |  |  |  | 0.183 |
| 0-3000 | 90（12.82） | 57（11.75） | 32（14.75） |  |  |
| 3000-4000 | 333（47.44） | 239（49.28） | 94（43.32） |  |  |
| >4000 | 249（35.47） | 185（38.14） | 64（29.49） |  |  |
| Smoking, n (%) |  |  |  | χ²=15.28 | **<0.001** |
| No | 630 (93.75) | 462 (96.05) | 168 (87.96) |  |  |
| Yes | 42 (6.25) | 19 (3.95) | 23 (12.04) |  |  |
| Drinking, n (%) |  |  |  | χ²=19.57 | **<0.001** |
| No | 638 (94.94) | 468 (97.30) | 170 (89.01) |  |  |
| Yes | 34 (5.06) | 13 (2.70) | 21 (10.99) |  |  |
| Take multiple medications, n (%) |  |  |  | χ²=0.70 | 0.402 |
| No | 41 (6.10) | 27 (5.61) | 14 (7.33) |  |  |
| Yes | 631 (93.90) | 454 (94.39) | 177 (92.67) |  |  |
| Systolic blood pressure (mm/Hg), Mean ± SD | 136.58 ± 17.00 | 136.57 ± 17.18 | 136.61 ± 16.59 | t=-0.03 | 0.978 |
| Diastolic blood pressure (mm/Hg), Mean ± SD | 76.08 ± 9.41 | 76.05 ± 9.39 | 76.17 ± 9.50 | t=-0.15 | 0.882 |
| Pulse (times/min), Mean ± SD | 75.83 ± 11.84 | 76.12 ± 11.68 | 75.08 ± 12.24 | t=1.03 | 0.305 |
| Recent hospitalization, n (%) |  |  |  | χ²=6.57 | **0.010** |
| No | 625 (93.01) | 455 (94.59) | 170 (89.01) |  |  |
| Yes | 47 (6.99) | 26 (5.41) | 21 (10.99) |  |  |
| Living condition, n (%) |  |  |  | χ²=1.47 | 0.480 |
| Live alone | 92 (13.69) | 69 (14.35) | 23 (12.04) |  |  |
| Living with children | 201 (29.91) | 138 (28.69) | 63 (32.98) |  |  |
| nursing homes | 379 (56.40) | 274 (56.96) | 105 (54.97) |  |  |
| Biochemical indicators and trace elements test | | | | | |
| Vitamin D (ng/ml), Mean ± SD | 23.13 ± 8.41 | 22.94 ± 8.53 | 23.60 ± 8.09 | t=-0.91 | 0.362 |
| Hemoglobin, Mean ± SD | 139.68 ± 13.39 | 140.03 ± 13.20 | 138.82 ± 13.84 | t=1.06 | 0.291 |
| Total protein, Mean ± SD | 73.91 ± 3.84 | 73.83 ± 3.73 | 74.12 ± 4.10 | t=-0.89 | 0.376 |
| Albumin, Mean ± SD | 44.98 ± 2.40 | 45.06 ± 2.30 | 44.77 ± 2.63 | t=1.31 | 0.190 |
| Globulin, Mean ± SD | 28.94 ± 3.23 | 28.77 ± 3.21 | 29.36 ± 3.26 | t=-2.13 | **0.033** |
| Total bilirubin, Mean ± SD | 18.90 ± 5.67 | 18.93 ± 5.85 | 18.82 ± 5.18 | t=0.24 | 0.812 |
| Alanine aminotransferase, M (Q₁, Q₃) | 17.00 (12.00, 22.00) | 16.00 (12.00, 23.00) | 17.00 (12.00, 21.00) | Z= -0.09 | 0.928 |
| Aspartate aminotransaminase, M (Q₁, Q₃) | 20.00 (17.00, 23.00) | 20.00 (17.00, 23.00) | 20.00 (17.00, 23.00) | Z= -0.38 | 0.705 |
| Creatinine, M (Q₁, Q₃) | 68.00 (59.00, 81.00) | 68.00 (59.00, 80.00) | 69.00 (59.00, 83.00) | Z= -0.44 | 0.659 |
| Uric acid, M (Q₁, Q₃) | 334.50 (287.00, 377.25) | 336.00 (285.00, 376.00) | 332.00 (288.50, 379.00) | Z= -0.21 | 0.830 |
| Calcium, Mean ± SD | 2.39 ± 0.07 | 2.39 ± 0.06 | 2.40 ± 0.08 | t=-1.37 | 0.170 |
| Magnesium, Mean ± SD | 0.89 ± 0.04 | 0.89 ± 0.04 | 0.89 ± 0.04 | t=0.58 | 0.561 |
| Phosphorus, Mean ± SD | 1.18 ± 0.11 | 1.18 ± 0.12 | 1.18 ± 0.08 | t= -0.72 | 0.473 |
| Total cholesterol, Mean ± SD | 5.24 ± 1.05 | 5.26 ± 1.06 | 5.19 ± 1.00 | t=0.84 | 0.402 |
| Triglyceride, M (Q₁, Q₃) | 1.59 ± 1.03 | 1.58 ± 1.09 | 1.61 ± 0.87 | t=-0.31 | 0.760 |
| High-density lipoprotein, Mean ± SD | 1.48 ± 0.35 | 1.49 ± 0.35 | 1.47 ± 0.34 | t=0.71 | 0.479 |
| Low-density lipoprotein, Mean ± SD | 2.85 ± 0.84 | 2.87 ± 0.85 | 2.80 ± 0.81 | t=0.94 | 0.350 |
| Fasting blood glucose, Mean ± SD | 5.86 ± 1.36 | 5.89 ± 1.38 | 5.78 ± 1.31 | t=0.98 | 0.330 |
| Fried’s Frailty Phenotype | | | | | |
| Exercise time (hours), n (%) |  |  |  | - | **<0.001** |
| 0 | 218 (32.44) | 139 (28.90) | 79 (41.36) |  |  |
| 1-2 | 380 (56.55) | 277 (57.59) | 103 (53.93) |  |  |
| 2-3 | 2 (0.30) | 2 (0.42) | 0 (0.00) |  |  |
| >3 | 72 (10.71) | 63 (13.10) | 9 (4.71) |  |  |
| Weight loss, n (%) |  |  |  | χ²=141.94 | **<0.001** |
| No | 620 (92.26) | 481 (100.00) | 139 (72.77) |  |  |
| Yes | 52 (7.74) | 0 (0.00) | 52 (27.23) |  |  |
| Decreased grip strength, n (%) |  |  |  | χ²=275.01 | **<0.001** |
| No | 571 (84.97) | 478 (99.38) | 93 (48.69) |  |  |
| Yes | 101 (15.03) | 3 (0.62) | 98 (51.31) |  |  |
| Decreased physical activity, n (%) |  |  |  | χ²=269.28 | **<0.001** |
| No | 575 (85.57) | 479 (99.58) | 96 (50.26) |  |  |
| Yes | 97 (14.43) | 2 (0.42) | 95 (49.74) |  |  |
| Often tired, n (%) |  |  |  | χ²=95.79 | **<0.001** |
| No | 636 (94.64) | 481 (100.00) | 155 (81.15) |  |  |
| Yes | 36 (5.36) | 0 (0.00) | 36 (18.85) |  |  |

**Note:**

Z: Mann-Whitney test, χ²: Chi-square test, -: Fisher exact

M: Median, Q₁: 1st Quartile, Q₃: 3st Quartile

M (Q₁, Q₃)：Median (M）, interquartile spacing (P25-P75)
